# Detection of Internal Defects in Concrete and Evaluation of a Healthy Part of Concrete by Noncontact Acoustic Inspection Using Normalized Spectral Entropy and Normalized SSE

**DOI:** 10.3390/e24020142

**Published:** 2022-01-18

**Authors:** Kazuko Sugimoto, Tsuneyoshi Sugimoto

**Affiliations:** Graduate School of Engineering, Toin University of Yokohama, 1614 Kurogane-cho, Aoba-ku, Yokohama 225-8503, Japan

**Keywords:** spectral entropy, spatial spectral entropy, SSE, non-destructive testing, defect detection, noncontact acoustic inspection

## Abstract

Non-destructive testing, with non-contact from a remote location, to detect and visualize internal defects in composite materials such as a concrete is desired. Therefore, a noncontact acoustic inspection method has been studied. In this method, the measurement surface is forced to vibrate by powerful aerial sound waves from a remote sound source, and the vibration state is measured by a laser Doppler vibrometer. The distribution of acoustic feature quantities (spectral entropy and vibrational energy ratio) is analyzed to statistically identify and evaluate healthy parts of concrete. If healthy parts in the measuring plane can be identified, the other part is considered to be internal defects or an abnormal measurement point. As a result, internal defects are detected. Spectral entropy (SE) was used to distinguish between defective parts and healthy parts. Furthermore, in order to distinguish between the resonance of a laser head and the resonance of the defective part of the concrete, spatial spectral entropy (SSE) was also used. SSE is an extension of the concept of SE to a two-dimensional measuring space. That is, based on the concept of SE, SSE is calculated, at each frequency, for spatial distribution of vibration velocity spectrum in the measuring plane. However, these two entropy values were used in unnormalized expressions. Therefore, although relative evaluation within the same measurement surface was possible, there was the issue that changes in the entropy value could not be evaluated in a unified manner in measurements under different conditions and environments. Therefore, this study verified whether it is possible to perform a unified evaluation for different defective parts of concrete specimen by using normalized SE and normalized SSE. From the experimental results using cavity defects and peeling defects, the detection and visualization of internal defects in concrete can be effectively carried out by the following two analysis methods. The first is using both the normalized SE and the evaluation of a healthy part of concrete. The second is the normalized SSE analysis that detects resonance frequency band of internal defects.

## 1. Introduction

Spectral entropy is often used in the time domain in recent years in the field of speech [1,2] and electroencephalography (EEG) analysis [3,4]. Spectral entropy is a feature that represents the whiteness of a signal. In information theory, entropy was first defined by Shannon and Weaver in 1949 [5] and further applied to the signal power spectrum by Johnson and Shore in 1884 [6]. Entropy used in this paper is based on Shannon’s entropy and applied to the frequency domain.

In recent years, collapse accidents due to the aging deterioration of concrete structures (tunnels, buildings, bridges, etc.) have occurred all over the world. At the inspection site of concrete structures, most of the on-site inspections are tap inspections and visual inspections. The tapping inspection largely depends on the skill and experience of the inspector, and there is a problem that it is expensive to install scaffolding in a high place, so it is expected to develop a method that can measure from a long distance without contact.

In order to solve this problem, a noncontact acoustic inspection method has been studying using acoustic irradiation induced vibration [7,8]. In this method, vibration energy is radiated to the entire surface by sound waves, and the vibration velocity distribution of the target surface is measured by a scanning laser Doppler vibrometer (SLDV). It has been clarified that cracks or cavity defects existing in the shallow layer of concrete can be detected even by the vibration of sound waves, because flexural resonance is likely to occur [9]. On the other hand, if the return light of the SLDV cannot be sufficiently obtained depending on the surface condition, an abnormal measurement point is observed and it is a problem at the time of measurement. However, since the vibration velocity spectrum measured in that case can be regarded as white noise, the defect detection algorithm [10] and the healthy part extraction algorithm [11] using the vibrational energy ratio [9] and the spectral entropy were devised. Further, since a scanning laser Doppler vibrometer (SLDV) for measuring the target surface two-dimensionally has high sensitivity, there is the issue that it is affected by strong reflected sound waves and causes a resonance phenomenon in the internal mechanism such as a Galvano mirror of a laser head. However, in a case of resonance of the SLDV, the same phenomenon occurs at all measurement points. Therefore, in order to distinguish it from the resonance phenomenon due to the defective part of concrete, spatial spectral entropy (SSE) [12] was devised that extended the concept of spectral entropy (SE) to a two-dimensional measuring space. By using SSE, it is possible to distinguish between the resonance due to the laser head of SLDV and the resonance of the defective part of concrete.

Conventionally, these two entropy amounts have been used without normalization. For that reason, although relative evaluation within the same measurement plane was possible, there was the issue that changes in the entropy value could not be evaluated in a unified manner in measurements under different conditions and environments. Therefore, in this paper, it is verified whether it is possible to perform a unified evaluation of different defective parts of concrete specimen using normalized SE and normalized SSE.

## 2. Experimental Method

**Figure 1:**
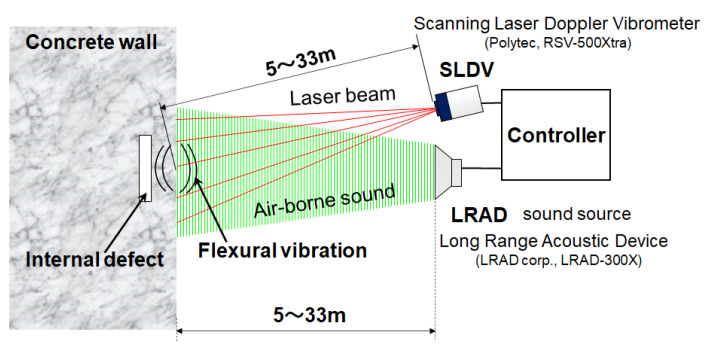
Experimental setup.

Figure 1 shows the fundamental experimental setup of the noncontact acoustic inspection method [7,8,9]. As a sound source, a long-range acoustic device (LRAD; Genasys Inc., San Diego, CA, USA; LRAD-300X) is used. In order to detect and visualize internal defects in composite materials from a distance, especially concrete, LRAD emits powerful aerial sound waves to the measuring plane. Vibration energy is given to the measuring plane, and the vibration velocity distribution of the two-dimensional surface is measured by a scanning laser Doppler vibrometer (SLDV; Polytec GmbH, Waldbronn, Germany; PSV-500Xtra). If there are internal defects (cracks, peeling, cavities, etc.), flexural vibration occurs, and the resonance vibration state is measured.

After performing “Time–Frequency gate” processing [8] to reduce noise and improve the signal-to-noise ratio, the vibration velocity spectrum is calculated, and FFT analysis is performed. As a result, internal defects are detected, and an acoustic defect image is created.

## 3. Normalization of Two Types of Entropy for Defect Detection

There are two methods to detect internal defects for noncontact acoustic inspection. One is a method to draw a scatter plot using two acoustic feature quantities (vibrational energy ratio and spectral entropy), and to identify a healthy part, a defective part, or an abnormal measurement point by the measured point’s existence area. The other is a method to detect the resonance frequency bands observed on the measurement surface by using spatial spectral entropy. These two methods have been applied individually but can be combined to perform internal defect detection in concrete.

### 3.1. Normalized Spectral Entropy

Spectral entropy (SE) is a feature quantity that expresses the whiteness of a signal. It has a high value for a signal with a uniform spectrum, such as white noise, and a low value for a signal with a non-uniform spectrum, such as an audio signal. The spectral entropy [10] previously used was expressed by the following equation.

(1) $$H=-\sum_{f}P_{f}\log_{2}P_{f}\;P_{f}=\frac{S_{f}}{\sum_{f}S_{f}}\;$$

In the previous paper, $S_{f}$ was defined as the amplitude spectrum of vibration velocity, but after that, the power spectrum of vibration velocity is more suitable as $S_{f}$ for entropy to detect defects. 

Normalized spectral entropy has been reported for signal analysis in the time domain [13,14]. For internal defect detection using entropy, spectral entropy has not been normalized, so normalized spectral entropy is introduced to verify the detection of internal defects. Normalized spectral entropy is defined by the following equation.

(2) $$H=-\frac{\sum_{f}P_{f}\log_{2}P_{f}}{\log_{2}\left\{N\left[f_{1},f_{2}\right]\right\}}\;P_{f}=\frac{S_{f}}{\sum_{f}S_{f}}\;$$

Here, $S_{f}$ is the power spectrum of the vibration velocity at a measurement point. $N\left[f_{1},f_{2}\right]$ is the number of sampling points in the vibration velocity spectrum at a measurement point. That is, N is the number of samples. The frequency range of the spectrum is selected as a frequency band of used emitted waves, in some cases, a frequency range to analyze.

### 3.2. Normalized Spatial Spectral Entropy (SSE)

**Figure 2:**
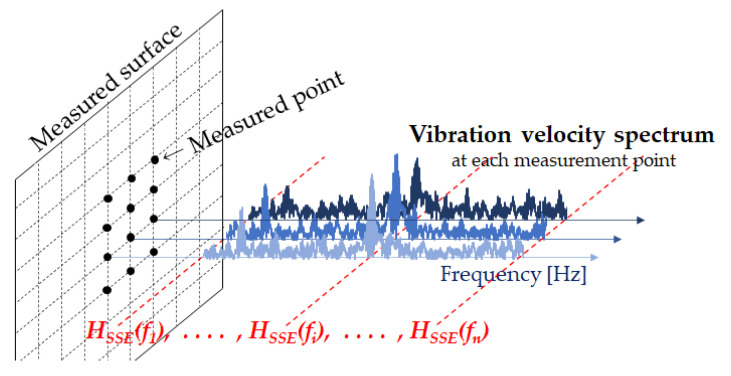
Conceptual diagram of SSE analysis.

Figure 2 shows a conceptual diagram of SSE analysis. Spatial spectral entropy (SSE) [12] is spectral entropy extended to space. In noncontact acoustic inspection, a power spectrum of vibration velocity observed at a measurement point is regarded as a probability distribution, and SE is calculated in the measured plane for each frequency. In this way, information entropy is calculated. Using each vibration velocity spectrum at all measured points, SSE analysis can detect not only the resonance frequency of internal defects of concrete but also the resonance frequency of a laser head of SLDV. When SSE was introduced, SSE was not normalized, but the normalized SSE needs to be defined. It is defined by the following equation.

(3) $$H_{SSE}\left(f\right)=-\frac{\sum_{i=1}^{m}\sum_{j=1}^{n}P_{i,j}\left(f\right)\log_{2}P_{i,j}\left(f\right)}{\log_{2}\left(m\cdot n\right)}\;P_{i,j}\left(f\right)=\frac{S_{i,j}\left(f\right)}{\sum_{i=1}^{m}\sum_{j=1}^{n}S_{i,j}\left(f\right)}$$

where $H_{SSE}\left(f\right)$ is spectral entropy that extended to real space and is a function of frequency f. $S_{i,j}\left(f\right)$ is the frequency f[Hz] component of the power spectrum obtained by discrete Fourier transforming a signal of vibration velocity measured at a measured point $r_{i,j}$. The subscript *i* and *j* are the grid point numbers for the vertical and horizontal direction, respectively. $P_{i,j}\left(f\right)$ is probability that $S_{i,j}\left(f\right)$ exists in the measured plane. Multiplying *m* by *n* gives the number of all measurement points.$\;H_{SSE}\left(f\right)$ indicates information entropy obtained by calculating the probability that the frequency f [Hz] component of vibration velocity spectrum exists in the measurement plane.

## 4. Why Normalize Spectral Entropy?

From the difference between spectral entropy and normalized spectral entropy, it is explained why it is necessary to normalize spectral entropy. Using the result of concrete specimen, it is explained how general defect detection is performed and how a healthy part of concrete is identified, and it becomes clear why the normalization of spectral entropy is necessary in the detection of internal defects of concrete.

### 4.1. Spectral Entropy and Normalized Spectral Entropy

**Figure 3:**
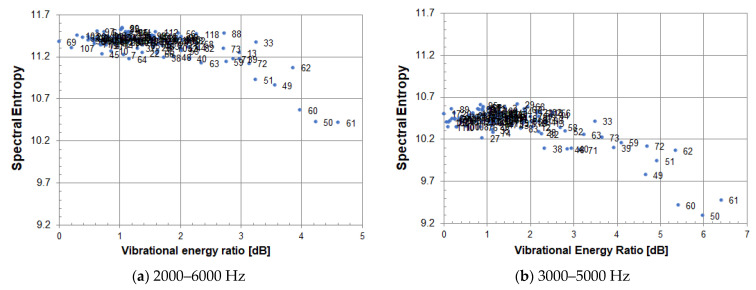
Scatter plot of spectral entropy and vibrational energy ratio. The analysis frequency range is (**a**) 2000–6000 Hz, (**b**) 3000–5000 Hz.

Figure 3 shows the experimental results for a circular cavity defect (Φ200, 60). The notation (Φ200, 60) indicates that the diameter of a circular cavity defect is 200 mm, and the embedded depth of the defect is 60 mm. From the measured vibration velocity distribution, the vibrational energy ratio and spectral entropy at each measured point was calculated. Figure 3 shows scatter plots of the vibrational energy ratio and spectral entropy. Data analysis is performed (a) in the frequency band of 2000–6000 Hz and (b) in that of 3000–5000 Hz. The analysis frequency bandwidths of (a) and (b) are 4000 Hz and 2000 Hz, respectively, and the sampling numbers in the frequency domains of (a) and (b) are 4400 and 2200, respectively. The values of spectral entropy take influence from sampling numbers and cannot be evaluated unifiedly as they are.

In general, the analysis frequency band is determined by the frequency band of the emitted sound waves, and the frequency band of the emitted sound waves is selected to include the resonance frequency band of internal defects. In order to compare data under different measurement conditions, it is necessary to eliminate the influence of the difference in the number of samplings due to different analysis frequency bands. The solution is to normalize.

**Figure 4:**
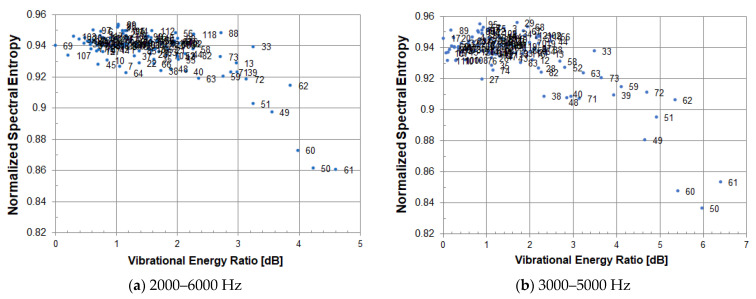
Scatter plot of normalized spectral entropy and vibrational energy ratio; Analysis frequency range is (**a**) 2000–6000 Hz, (**b**) 3000–5000 Hz.

If normalized spectral entropy is used, it is possible to evaluate on the same scale. (a) and (b) in Figure 4 are the normalized figures of (a) and (b) in Figure 3, respectively. The distribution of a healthy part of concrete is at the same level. It is possible to distinguish between a healthy part of concrete and a defective part on the same scale.

### 4.2. General Defect Detection and Normalized Spectral Entropy

**Figure 5:**
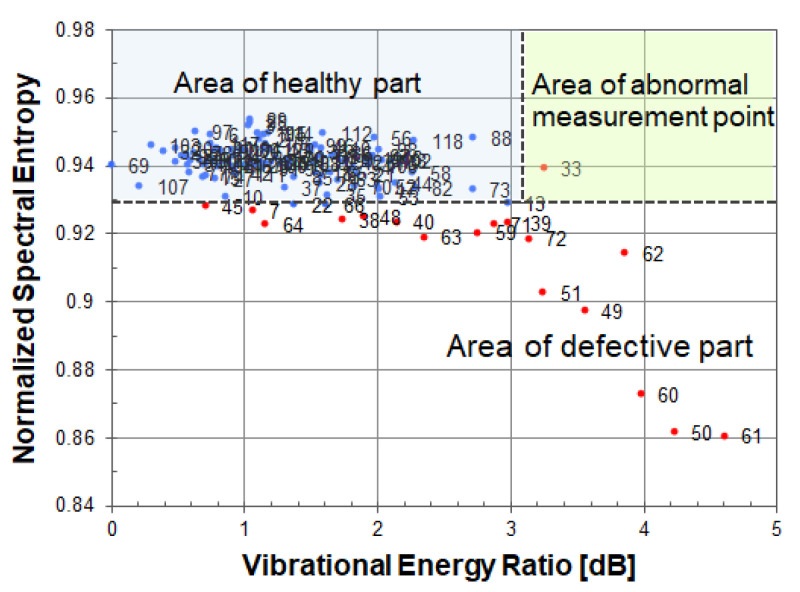
Scatter diagram of the normalized spectral entropy versus vibrational energy ratio.

As shown in Figure 5, if a scatter plot is drawn using two acoustic feature quantities (vibrational energy ratio and spectral entropy), it is possible by the measurement point’s existence area to identify a point of a healthy part, a point of a defective part, or an abnormal measurement point. The figure is obtained from the measured result of the vibration velocity distribution using a cavity defect (Φ200, 60). The notation (Φ200, 60) indicates that the diameter of a circular cavity defect is 200 mm, and the embedded depth is 60 mm. In Figure 5, three areas of a healthy part, a defective part, and an abnormal measurement point can be seen. These areas are separated by a black dotted line. In the Figure, the measured points of a healthy part are dotted in blue, and the other measured points such as a defective part and an abnormal measurement point are dotted in red.

In general, there are the following tendencies: the healthy part has low vibration energy and high spectral entropy; the defective part has high vibration energy and low spectral entropy; and an abnormal measurement point has high vibration energy and high spectral entropy. This is summarized in Table 1.

### 4.3. Healthy Part Identification and Normalized Spectral Entropy

In defect detection, it is necessary to identify a healthy part of concrete in the measurement plane. If a healthy part can be identified, the other parts are the defective part or an abnormal measurement point.

**Figure 6:**
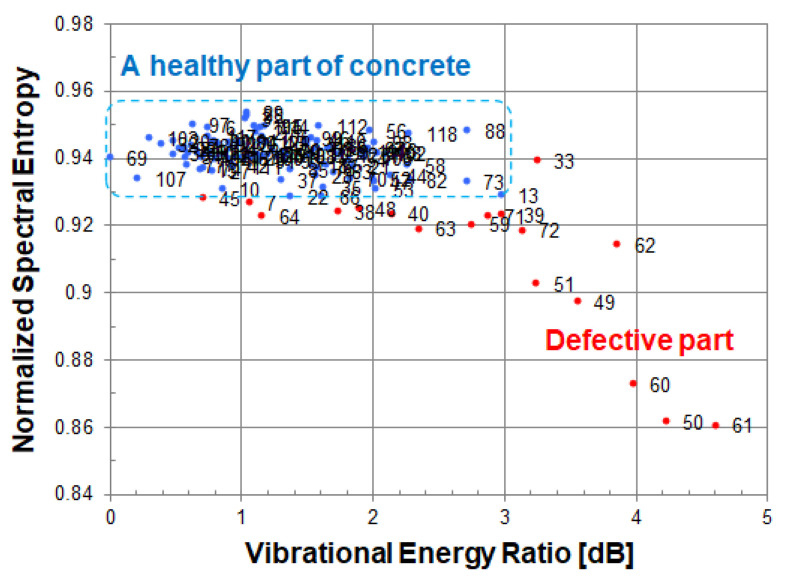
Scatter diagram of normalized spectral entropy versus vibrational energy ratio.

For one measurement point, two acoustic feature quantities (vibrational energy ratio and spectral entropy) are calculated from its vibration velocity spectrum. It has been found that each distribution of two acoustic feature quantities in a healthy part of concrete follow the normal distribution. Taking advantage of this fact, measuring points in a healthy part can be found by statistical methods. Using the healthy part extraction algorithm [11], a healthy part of concrete can be identified as shown in Figure 6. Since it is not necessary to divide the three areas by a straight line in this method, it can be effectively applied to a real concrete structure. If the value of the spectral entropy at a measured point is smaller than the distribution of a healthy part of concrete, it is highly likely that it is a defective part, and if the value of the spectral entropy is larger than that, it may be an abnormal measurement point.

In defect detection, a defective part and a healthy part are discriminated based on the distribution of a healthy part of concrete. So, defect detection will be robust if the distribution of spectral entropy at a healthy part shows the same level value without depending on the measuring conditions. It is possible to compare the measurement results of different actual concrete structures with a unified standard.

## 5. Why Normalize Spatial Spectral Entropy (SSE)?

### Spatial Spectral Entropy and Normalized Spatial Spectral Entropy

**Figure 7:**
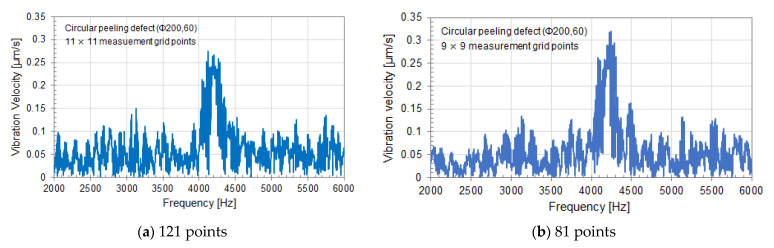
Vibration velocity spectrum at the center of a circular peeling defect (Φ200, 60). The measurement grid points are (**a**) 121 (11 × 11) points and (**b**) 81(9 × 9) points. The measured point number is (**a**) 61, (**b**) 41.

Figure 7 shows the experimental results for a circular peeling defect (Φ200, 60). The notation (Φ200, 60) indicates that the diameter of a circular peeling defect is 200 mm, and the embedded depth is 60 mm. Figure 7 shows the vibration velocity spectrum at the center of the circular peeling defect (Φ200, 60). The measurement grid points are 121 (11 × 11) points for (a) and 81 (9 × 9) points for (b). The SSE value depends on the number of measurement grid points due to the equation of SSE analysis. So, comparison is performed using the same circular defect with different measurement grid points. Comparing the vibration velocity spectra at the center of the circular peeling defect in (a) and (b), the frequency of the resonance peak due to a circular peel defect does not change, and the magnitude of the peak is almost the same level. The level of vibration velocity in a healthy part of concrete is about the same.

**Figure 8:**
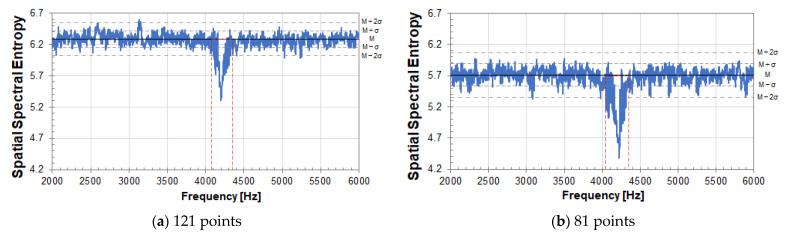
SSE analysis result of a circular peeling defect (Φ200, 60). The measurement grid points are (**a**) 121 (11 × 11) points and (**b**) 81(9 × 9) points.

**Figure 9:**
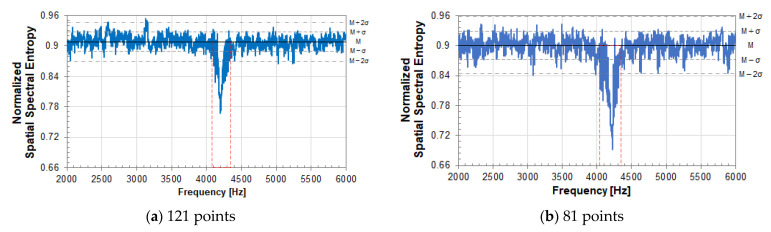
Normalized SSE analysis result of a circular peeling defect (Φ200, 60); Measurement grid points is (**a**) 121 (11 × 11) points, (**b**) 81(9 × 9) points.

Figure 8 and Figure 9 show the results of SSE analysis for a circular peeling defect (Φ200, 60). The measurement grid points are 121 (11×11) points for (a) and 81 (9×9) points for (b). The resonance frequency of an internal defect in the measurement plane can be detected by SSE analysis. That is, the resonance frequency band of a circular peeling defect in Figure 7 is detected by SSE analysis in Figure 8 and Figure 9, so that the SSE value decreases. In the case of a healthy part of concrete, the SSE value takes a value that fluctuates around the median of its fluctuation.

In Figure 8, the median of SSE value fluctuation is 6.28 in (a) and 5.71 in (b). M in the figure indicates the median of SSE value fluctuation, and σ indicates its standard deviation. The standard deviation σ is 0.13 in (a) and 0.18 in (b). The larger the number of measurement grid points, the larger the SSE value. The number of measurement grid points on the measurement plane corresponds to the number of data when calculating SSE by equation (3), and SSE value is affected to the number.

Therefore, in Equation (3), the SSE is normalized to eliminate the influence of the number of data, that is, the number of measurement grid points. In Figure 9, the median M of the normalized SSE value fluctuation is 0.908 in (a) and 0.901 in (b). σ is 0.019 in (a) and 0.029 in (b). According to the result of normalized SSE, it is expected that the value of SSE can be evaluated on the same scale under different measurement conditions regardless of the number of measurement grid points. From this result, the resonance frequency band, surrounded by the red dotted line frame, due to a circular peeling defect is the same in Figure 8 and Figure 9. Therefore, it is not affected by the number of measurement grid points.

## 6. Effects of Normalization by Two Types of Internal Defects

The effect of normalization was investigated for two types of defects, such as a cavity and a peeling as an internal defect of concrete.

### 6.1. Cavity Defect and Peeling Defect

**Figure 10:**
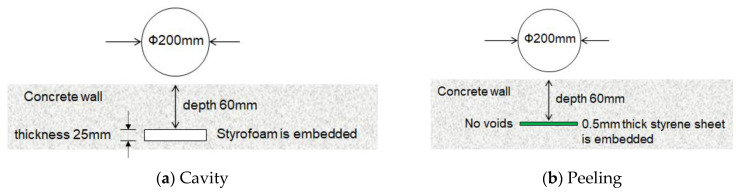
Shape and embedded depth of a circular defect; (**a**) cavity, (**b**) peeling.

The concrete wall specimen has a size of 2000 mm × 2000 mm × 300 mm and a weight of approximately 1200 kg. The shape and embedded depth of a circular cavity defect are as shown in Figure 10a. When the concrete was poured, Styrofoam with a thickness of 25 mm was embedded to simulate a cavity defect. The shape and embedded depth of a circular peeling defect are as shown in Figure 10b. When concrete was poured, a styrene sheet with a thickness of 0.5 mm was embedded to simulate a defect such as a crack and a peeling.

### 6.2. Measurement Conditions

The concrete wall with a cavity defect (Φ200, 60) or a peeling defect (Φ200, 60) was installed about 5.0 m from an LRAD of sound source. The distance from a laser Doppler vibrometer to the measurement surface is approximately 7.7 m for the cavity defect (Φ200, 60), 8.0 m for the peeling defect (Φ200, 60). The notation (Φ200, 60) indicates that the diameter of a circular defect is 200 mm, and the embedded depth is 60 mm. As an emitted waveform, a single-tone burst wave [8,15] (frequency range 2000–6000 Hz, modulation frequency 200 Hz, interval time 50 ms, pulse width 3 ms, overall waveform length 1050 ms) was used. The signal of the vibration velocity at one measurement point was measured 5 times. In order to realize stable laser measurement, the median value of five signals, which were measured repeatedly, was adopted as an average value. The measured grid points were 121 (11 × 11) points in one measurement. The vertical or horizontal measurement intervals for the cavity defect (Φ200, 60) were both approximately 3.7 cm. The vertical and horizontal measurement intervals for the peeling defect (Φ200, 60) were both approximately 3.9 cm.

### 6.3. Vibration Velocity Spectrum in the Center of Circular Defect

**Figure 11:**
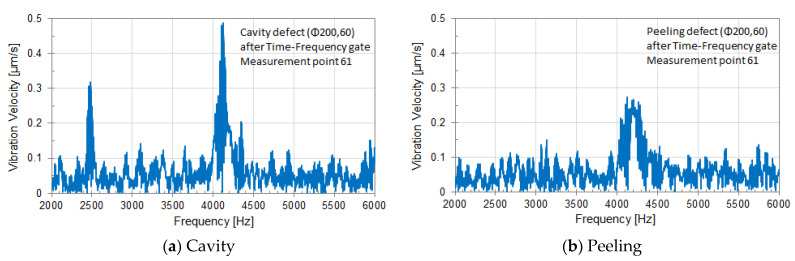
Vibration velocity spectrum at measurement point 61 near the center of a circular defect; (**a**) cavity, (**b**) Peeling.

Figure 11 shows the vibration velocity spectrum at measurement point 61 in almost the center of a circular defect. (a) and (b) are the results obtained from each experiment of the circular cavity defect (Φ200, 60) and the circular peeling defect (Φ200, 60), respectively. Each vibration velocity spectrum in the frequency domain was calculated by FFT from the target signal extracted using Time-Frequency gate [8].

The time–frequency gate makes good use of the propagation time difference between light and sound waves to extract the target signal. The sound wave is intermittent and its frequency changes stepwise. Therefore, the vibration is performed at a specific frequency at a specific time. That is, when the time zone in which the sound waves make the concrete surface vibrating and emission frequency band at that time are known, the target signal can be extracted in the time domain and the frequency domain respectively. As a result, it is possible to reduce the influence of reflected waves on the high-sensitivity scanning laser Doppler vibrometer by the direct wave from the sound source, the reflected wave from the concrete surface, and the reverberation from the surroundings.

Since the frequency range of the emitted waveform is 2000 to 6000 Hz, the analysis frequency range was set to the same frequency range, and the FFT was calculated in that frequency range.

In Figure 11a, a sharp peak due to a circular cavity defect (Φ200, 60) is seen at about 4100 Hz. Moreover, a small peak, that seems to be the resonance of a laser head, is seen at around 2500 Hz. Since this method uses strong sound waves, resonance may occur in the mechanism such as the Galvano mirror of a laser head, and resonance peaks may be seen. Such resonance peaks due to a laser head may vary in magnitude depending on the arrangement of a sound source and a laser Doppler vibrometer and the surrounding environment including the measurement surface. In a case, such a peak may not emerge.

In Figure 11b, a broad peak due to a circular peeling defect (Φ200, 60) can be seen at around 4200 Hz. The circular cavity defect (Φ200, 60) has a sharper peak and a larger amplitude than the circular peeling defect (Φ200, 60).

### 6.4. Scatter Diagram of Normalized Spectral Entropy vs. Vibrational Energy Ratio

**Figure 12:**
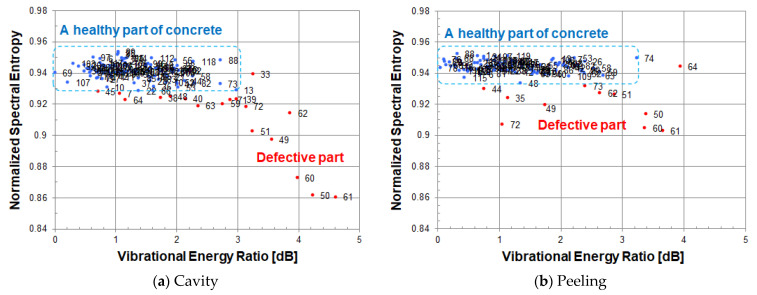
Scatter diagram of normalized spectral entropy versus vibrational energy ratio; (**a**) cavity, (**b**) peeling.

At each measured point, the vibrational energy ratio and spectral entropy are calculated from its vibration velocity spectrum. As shown in Figure 12, when looking at a scatter plot based on spectral entropy and vibrational energy ratio, many measured points in a healthy part are gathered in the upper left, and the remaining measurement points of the defective part are scattered while descending to the lower right.

In the scatter diagram, red or blue points indicate measured points, and the number to the right of its point is a measurement grid point number. The blue points are the measured points judged to be a healthy part of concrete, and the red points are the measured points including defective part or an abnormal measurement point.

Figure 12a,b use the same energy reference value (1.01 × 10^−11^ [m/s]^2^) when calculating the vibration energy ratio. As the energy reference value of the vibration energy ratio, the minimum value of vibration energy in the circular cavity defect (Φ200, 60) was adopted.

Since spectral entropy is normalized, a cavity defect and a peel defect can be compared on the same scale. A healthy part of concrete can be evaluated by normalized spectral entropy and vibrational energy ratio on the same scale. The value of spectral entropy in a healthy part of concrete is distributed in the range of 0.93–0.95. The value of vibration energy ratio in a healthy part of concrete is distributed in the range of 0–3 dB.

In a cavity defect, the measurement points of the defective part are scattered in the lower right direction from the distribution of a healthy part of concrete. On the other hand, in a peeling defect, since the vibration energy of the defective part is about the same as that of a healthy part of concrete, the measurement points are scattered below a healthy part of concrete. In order to distinguish between a healthy part and the defective part of peeling, it is necessary to evaluate by normalized spectral entropy.

### 6.5. Acoustic Defect Image with Evaluation of a Healthy Part of Concrete

**Figure 13:**
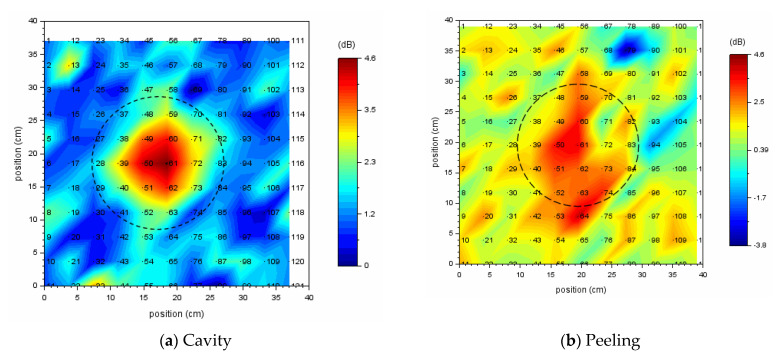
Acoustic defect image for a circular defect; (**a**) cavity, (**b**) peeling.

**Figure 14:**
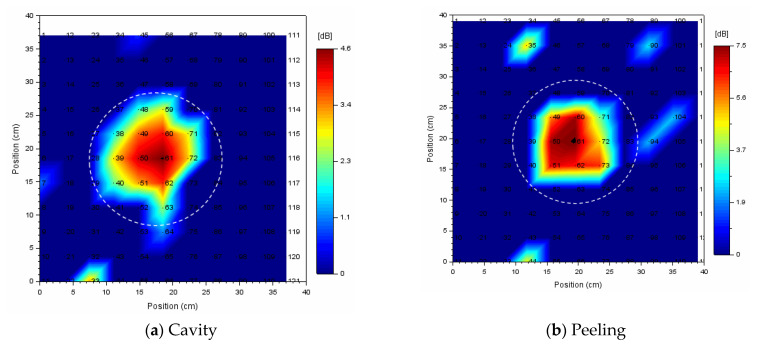
Acoustic defect image for a circular defect (Φ200, 60); (**a**) cavity, (**b**) peeling. The vibrational energy ratio in a healthy part of concrete is set to 0 dB and displayed.

Figure 13 shows acoustic defect images. Those images are represented by the vibrational energy ratio (see Figure 12). The black or white dotted circle in Figure 13 and Figure 14 shows the exact size and approximate location of a circular defect. In the acoustic defect images, dots indicate measurement grid points, and the number to the right of its dot is a measurement grid point number. Figure 13a was visualized using vibrational energy ratio at each measurement point for a circular cavity defect (Φ200, 60) (See Figure 12a). Figure 13b was visualized using vibrational energy ratio at each measurement point for a circular peeling defect (Φ200, 60) (See Figure 12b).

Comparing (a) and (b) in Figure 13, in the case of the cavity defect, the defect can be sufficiently identified by an acoustic image obtained by simply calculating the vibration energy ratio in the emission frequency band. But in the case of peeling defect, it is difficult to distinguish between a healthy part and a defect part. This is because, as shown in Figure 12b, the levels of the vibration energy of a healthy part and the defective part are almost the same, so that the defective part is buried in a healthy part in the acoustic image.

Therefore, in order to distinguish between a healthy part and the defective parts, it is important to evaluate a healthy part on the second axes called spectral entropy. In order to cause resonance and efficiently vibrate the defective parts, the optimum emitted waveform is selected depending on the type, size, and buried depth of internal defects, so the measurement conditions differ. In particular, the frequency range of the emitted waveform affects the spectral entropy value. By normalizing spectral entropy, this problem can be resolved. It is possible to accumulate and utilize data on a healthy part of concrete on the same scale even if the measurement conditions are different.

It is identified statistically whether each measured point is a healthy part of concrete or not using the healthy part extraction algorithm. In Figure 14, the vibrational energy ratio of a measured point identified as a healthy part of concrete is set to 0 dB and displayed. By doing so, the fluctuation noise at a healthy part is eliminated, the defective part is emphasized, and the defect image becomes easier to see.

In this way, robust defect detection can be performed by evaluating the distribution of a healthy part of concrete using normalized spectral entropy and vibrational energy ratio. Moreover, it is also applicable to actual concrete structures in various environments.

### 6.6. Normalized SSE Analysis

For a circular defect (Φ200, 60), a vibration velocity signal was observed at each measurement grid point by a scanning laser Doppler vibrometer (SLDV). After Time-Frequency gate processing, those signals were transformed to vibration velocity spectrum by FFT. Using 121 spectra obtained at each measurement grid point, SSE analysis was performed using Equation (3).

**Figure 15:**
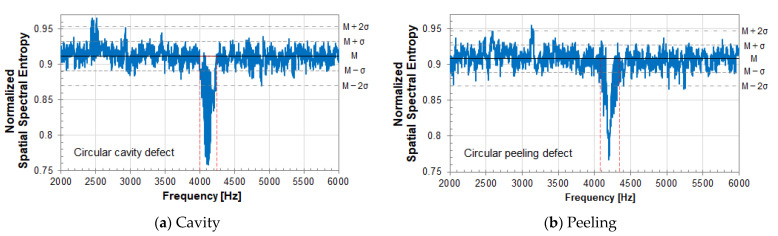
Result of SSE analysis for a circular defect (Φ200,60); (**a**) cavity, (**b**) peeling.

Figure 15 shows the result of SSE analysis. In the figure, the vertical axis is the SSE value, and the horizontal axis is the frequency. The red frame with a decreasing SSE value shows the resonance frequency band due to an internal defect. The SSE value tends to decrease at the resonance frequency of internal defects in concrete. In Figure 15, (a) represents a circular cavity defect (Φ200, 60), and (b) represents a circular peeling defect (Φ200, 60). M on the right side of the Figure indicates the median fluctuation of the SSE value, and σ indicates its standard deviation. In (a), M is 0.912, and σ is 0.02. In (b), M is 0.908, and σ is 0.019.

As a result of SSE analysis, the SSE value in a healthy part of concrete shows fluctuation in a certain range with respect to the median value of the distribution of SSE values. By normalizing the SSE, different internal defects can be evaluated on the same scale.

Suppose you have glasses that can see a specific frequency, such as infrared rays. When looking at the measurement surface, a local peak can be seen on the measurement surface. In that case, the SSE value decreases at that frequency. In other words, in SSE analysis, it is possible to detect a local peak on the measurement surface in the frequency domain. In particular, when a resonance peak due to an internal defect appears on the measurement plane, the SSE value decreases at that resonance frequency. When it has a uniform distribution or a normal distribution on the measurement surface, such as the case of resonance of a laser head, the SSE value rises according to the nature of entropy. The detected resonance frequency of a laser head is excluded from the calculation of the vibrational energy ratio.

**Figure 16:**
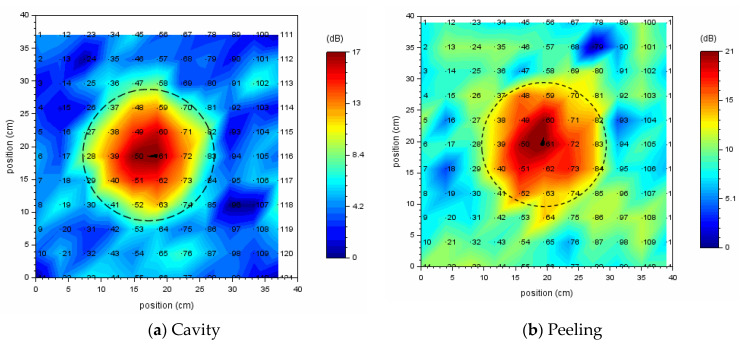
Acoustic defect image for a circular defect (Φ200, 60); (**a**) cavity, (**b**) peeling. The analysis frequency range for calculating the vibrational energy ratio is (**a**) 4000–4250 Hz and (**b**) 4120–4310 Hz.

By SSE analysis, both the resonance frequency bands of a cavity defect and a peeling defect were detected. As shown in Figure 16, acoustic defect images are created by vibrational energy ratio, which is calculated using the detected resonance frequency band as an analysis frequency range. (a) is a cavity defect, and the analysis frequency range is 4000–4250 Hz. (b) is a peeling defect, and the analysis frequency range is 4120–4310 Hz.

The black dotted circle in Figure 16 shows the exact size and approximate location of a circular defect. In the acoustic defect image, dots indicate measured grid points, and the number to the right of its dot is a measurement grid point number.

If the resonance frequency band of the internal defect is known by SSE analysis, a clear acoustic image of the internal defect can be obtained simply by calculating the vibrational energy ratio at the range of the resonance frequency band.

**Figure 17:**
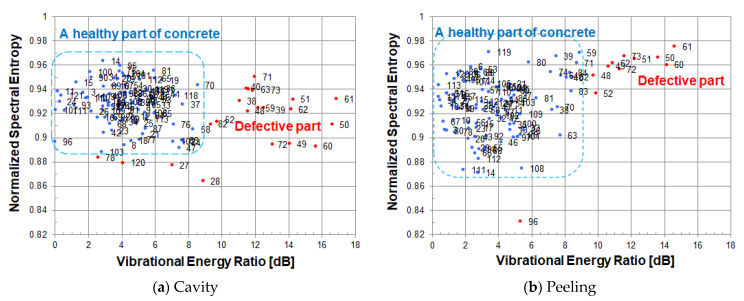
Scatter diagram of the normalized spectral entropy and vibrational energy ratio for a circular defect (Φ200, 60); (**a**) cavity, (**b**) peeling.

Figure 17 is a scatter diagram of normalized spectral entropy versus vibrational energy ratio calculated in each resonance frequency band. A measurement point in a healthy part is indicated by a blue dot, and a measurement point of the defective part is indicated by a red dot. The vibration energy of measurement points in the defective part is larger than that of measurement points in a healthy part.

Figure 17a,b use the same energy reference value (2.75 × 10^−13^ [m/s]^2^) when calculating the vibration energy ratio. As an energy reference value of the vibration energy ratio, the minimum value of vibration energy in the circular cavity defect (Φ200, 60) was adopted.

Vibration energy is calculated within the range of frequency band where resonance occurs, so the vibration energy at measurement points where the internal defect exist becomes large, and the defect image becomes clear.

## 7. Conclusions

Normalization is defined for two types of entropy used in noncontact acoustic inspection method and experimental and analysis results are showed for internal defect such as a cavity or a peeling. One is spectral entropy (SE) used to detect the internal defects of concrete in a non-destructive test. SE can also be used to evaluate a healthy part of concrete. The other is SSE (spatial spectral entropy), which can detect the resonance frequency band of internal defects in the measuring plane. Through two kinds of entropy, the effective detection and visualization of internal defects in concrete was verified. Each entropy can be used separately or together, depending on a situation of concrete structures.

## Figures and Tables

**Table 1:** Defect detection algorithm judgement.

Judgement	Vibrational Energy Ratio	Spectral Entropy
Healthy part	low	high
Defective part	high	low
Abnormal measurement point	high	high

## References

[1] Shen J., Hung J., Lee J. Robust entropy-based endpoint detection for speech recognition in noisy environments. Proceedings of the 5th International Conference on Spoken Language.

[2] Renevey P., Drygajlo A. Entropy Based Voice Activity Detection in Very Noisy Conditions. Proceedings of the EUROSPEECH 2001 Scandinavia, 7th European Conf. on Speech Communication and Technology.

[3] Inouye T., Shinosaki K., Sakamoto H. (1991). Quantification of EEG irregularity by use of the entropy of the power spectrum. Electroencephalogr. Clin. Neurophysiol..

[4] Rezek A., Roberts S.J. (1998). Stochastic complexity measures for physiological signal analysis. IEEE Trans. Biomed. Eng..

[5] Shannon C.E. (1948). A mathematical theory of communication. Bell Syst. Tech. J..

[6] Johnson R.W., Shore J.E. (1984). Which is the better entropy expression for speech processing, -S log S or log S?. IEEE Trans. Acoust.

[7] Akamatsu R., Sugimoto T., Utagawa N., Katakura K. (2013). Proposal of non contact inspection method for concrete structures using high-power directional sound source and scanning laser Doppler vibrometer. Jpn. J. Appl. Phys..

[8] Akamatsu R., Sugimoto T., Utagawa N., Katakura K. Study on Non contact acoustic imaging method for concrete structures –improvement of signal to-noise ratio by using tone burst wave method. Proceedings of the IEEE International Ultrasonics Symposium.

[9] Katakura K., Akamatsu R., Sugimoto T., Utagawa N. (2014). Study on detectable size and depth of defects in noncontact acoustic inspection method. Jpn. J. Appl. Phys..

[10] Sugimoto K., Akamatsu R., Sugimoto T., Utagawa N., Kuroda C., Katakura K. (2015). Defect-detection algorithm for noncontact acoustic inspection using spectrum entropy. Jpn. J. Appl. Phys..

[11] Sugimoto K., Sugimoto T., Utagawa N., Kuroda C., Kawakami A. (2018). Detection of internal defects of concrete structures based on statistical evaluation of healthy part of concrete by the noncontact acoustic inspection method. Jpn. J. Appl. Phys..

[12] Sugimoto K., Sugimoto T., Utagawa N., Kuroda C. (2019). Detection of resonance frequency of both the internal defects of concrete and the laser head of a laser Doppler vibrometer by spatial spectral entropy for noncontact acoustic inspection. Jpn. J. Appl. Phys..

[13] Viertio-Oja H., Maja V., Talja P., Tenkanen N., Tolvanen-Laakso H., Paloheimo M., Vakkuri A., Yli-Hankala A., Meriläinen P. (2004). Description of the Entropy^TM^ algorithm as applied in the Datex-Ohmeda S/5^TM^ entropy module. Acta Anaesthethesiol. Scand..

[14] Zaccarelli N., Li B.-L., Petrosillo I., Zurlini G. (2011). Order and disorder in ecological time-series: Introducing normalized spectral entropy. Ecol. Indicat..

[15] Sugimoto T., Sugimoto K., Kosuge N., Utagawa N. (2017). High-speed noncontact acoustic inspection method for civil engineering structure using multitone burst wave. Jpn. J. Appl. Phys..

